# Tocotrienols Prevent the Decline of Learning Ability in High-Fat, High-Sucrose Diet-Fed C57BL/6 Mice

**DOI:** 10.3390/ijms25063561

**Published:** 2024-03-21

**Authors:** Yugo Kato, Junhyoku Ben, Atsuto Noto, Shuntaro Kashiwaya, Yoshinori Aoki, Nobuo Watanabe, Hiroki Tsumoto, Yuri Miura, Koji Fukui

**Affiliations:** 1 Molecular Cell Biology Laboratory, Department of Bioscience and Engineering, College of Systems Engineering and Sciences, Shibaura Institute of Technology, Saitama 337-8570, Japan; yugo@tottori-u.ac.jp (Y.K.); mf22114@shibaura-it.ac.jp (J.B.); bn19015@shibaura-it.ac.jp (S.K.); 2 Department of Pathophysiological and Therapeutic Science, Tottori University, Yonago 683-8503, Japan; 3 Mitsubishi Chemical Corporation, Chiyoda, Tokyo 100-8251, Japan; 4 Biofluid Science and Engineering Laboratory, Department of Bioscience and Engineering, College of Systems Engineering and Sciences, Shibaura Institute of Technology, Saitama 337-8570, Japan; nobuo@sic.shibaura-it.ac.jp; 5 Research Team for Mechanism of Aging, Tokyo Metropolitan Institute for Geriatrics and Gerontology, Itabashi-ku, Tokyo 173-0015, Japan

**Keywords:** vitamin E, antioxidant, cognition, obesity

## Abstract

Obesity has been increasing worldwide and is well-known as a risk factor for cognitive decline. It has been reported that oxidative stress in the brain is deeply involved in cognitive dysfunction in rodent models. While there are many studies on oxidation in the liver and adipose tissue of obese mice, the relationship between obesity-induced cognitive dysfunction and brain oxidation has not been elucidated. Here, we show that obesity induced by a high-fat, high-sucrose diet (HFSD) alters cognitive function in C57BL/6 male mice, and it may involve the acceleration of brain oxidation. Tocotrienols (T3s), which are members of the vitamin E family, can prevent HFSD-induced cognitive changes. To elucidate these mechanisms, respiratory metabolism, locomotor activity, temperature around brown adipose tissue, and protein profiles in the cerebrum cortex were measured. Contrary to our expectation, respiratory metabolism was decreased, and temperature around brown adipose tissue was increased in the feeding of HFSD. The proteins that regulate redox balance did not significantly change, but 12 proteins, which were changed by HFSD feeding and not changed by T3s-treated HFSD compared to control mice, were identified. Our results indicated that HFSD-induced obesity decreases mouse learning ability and that T3s prevent its change. Additionally, feeding of HFSD significantly increased brain oxidation. However, further study is needed to elucidate the mechanisms of change in oxidative stress in the brain by obesity.

## 1. Introduction

Dementia-related conditions such as Alzheimer’s (AD) and Parkinson’s disease are associated with significant cognitive decline [1,2]. The mechanisms underlying cognitive dysfunction in conditions such as AD are highly complex. AD is characterized by amyloid-beta deposition and neurofibrillary tangles formed by hyperphosphorylated-tau protein in the brain [3]. These pathological features are disease-specific but accelerated oxidative stress and altered neurotrophic factor expression are well-known common pathologies in cognitive impairment [4,5]. Studies have shown that levels of lipid peroxides and nitrated proteins are increased in the brain of AD-model mice [6,7]. Other studies have demonstrated that the expression of neurotrophic factors such as nerve growth factor (NGF) and brain-derived neurotrophic factor (BDNF) is downregulated in the brain of AD model mice [8,9].

Between 1975 and 2016, the average body mass index increased worldwide [10]. It is anticipated that the prevalence of obesity will increase due to the decrease in movement opportunities during the COVID-19 pandemic. Evidence suggests that obesity increases the risk of cognitive decline [11,12,13]. Previous studies have demonstrated that obesity is associated with oxidative stress [14]. However, many studies have focused on the tissues that are closely related to the development of obesity-induced diseases such as cardiovascular disease and diabetes, and there is less direct evidence that obesity induces oxidation in the brain [15,16,17].

Research in 2015 and 2016 showed that tocotrienols (T3s) exhibit anti-obesity and neuroprotective functions [18,19]. T3s are vitamin E isoforms, as are tocopherols (TOCs). TOCs and T3s are classified by the presence or absence of double bonds on the side chain. Additionally, there are four types (α-, β-, γ-, and δ-) based on the position and number of methyl groups on the chroman ring [20]. A common biological function of vitamin E is antioxidant activity [20]. As T3s have unsaturated side chains, they more readily enter cells and exhibit greater antioxidant capacity than TOCs [21]. To exert antioxidant effects in the brain, T3s have to pass through the blood–brain barrier. Previously, we demonstrated that T3s can be detected in perfused brain tissues using high-performance liquid chromatography. That research also showed that the tight junctions were not broken [22]. Many studies have thus examined T3s as a means of attenuating cognitive decline [23,24,25]. However, the role of T3s in obesity-induced cognitive decline is still poorly understood.

For the present study, we hypothesized that diet-induced obesity in mice might alter cognitive function by exacerbating oxidative stress. Here, we investigated the learning ability of mice fed a high-fat, high-sucrose diet (HFSD) and T3s treatment compared to control mice through the Morris water maze test and Y-maze short-term memory. Moreover, we recorded the levels of 3-nitrotyrosine (3-NT, an index of oxidative stress in a broad sense) in the cerebrum cortex (Cortex) of HFSD-fed mice and performed a proteomic analysis of the Cortex to identify proteins significantly changed in expression by HFSD-induced obesity and T3s treatment. 

## 2. Results

### 2.1. HFSD Feeding Induced Obesity in C57BL/6 Mice

**Figure 1:**
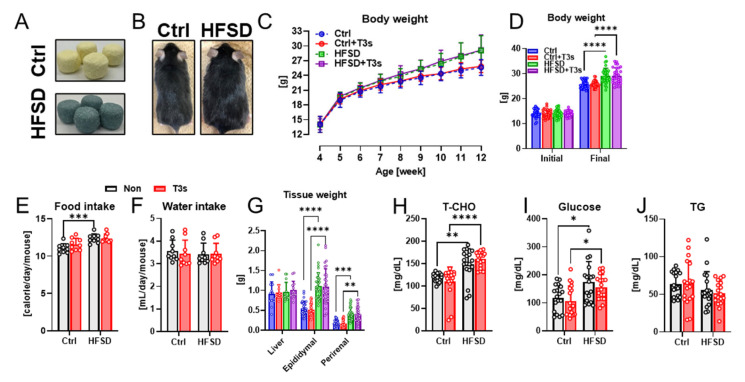
Characterization of HFSD-fed obese model mice. (**A**,**B**) Representative images of experimental diets and mice (12 weeks old). (**C**) Body weight changes during the treatment period are shown. (**D**) Initial and final body weight in duration (**** *p* < 0.0001, each experimental group; *n* = 31). (**E**,**F**) Average calorie and water intake (*** *p* < 0.001). (**G**) Weight of the liver (*n* = 13) and fat tissue (epididymal and perirenal; *n* = 31) at the time mice were dissected (** *p* < 0.01, *** *p* < 0.001, **** *p* < 0.0001). (**H**–**J**) Parameters of serum collected from the inferior vena cava (* *p* < 0.05, ** *p* < 0.01, **** *p* < 0.0001, all experimental groups and all parameters; *n* = 18). All data are shown as the mean ± SD.

To investigate the anti-obesity effects of T3s, the body weight of all mice was measured once per week. The body weight of all mice gradually increased during the feeding of each experimental diet (Figure 1C). While the body weight of HFSD-fed mice was significantly higher than that of the control diet (Ctrl)-fed mice, no significant differences were found between the body weight of HFSD-fed mice and that of HFSD + T3s-fed mice (Figure 1D). In both Ctrl and HFSD mice, no significant differences were found in calorie and water intake in the presence or absence of T3s (Figure 1E,F). Similarly, the epididymal and perirenal fat weight of both HFSD− and HFSD + T3s-fed mice were significantly greater than that of control mice (Figure 1G). Total cholesterol (T-CHO) and glucose levels were significantly elevated by feeding with HFSD and HFSD + T3s, respectively (Figure 1H,I). On the other hand, the concentration of Triglyceride (TG) was not altered by HFSD and/or T3s (Figure 1J).

### 2.2. Cognitive and Redox Balance Changes following Treatment with HFSD and/or T3s

**Figure 2:**
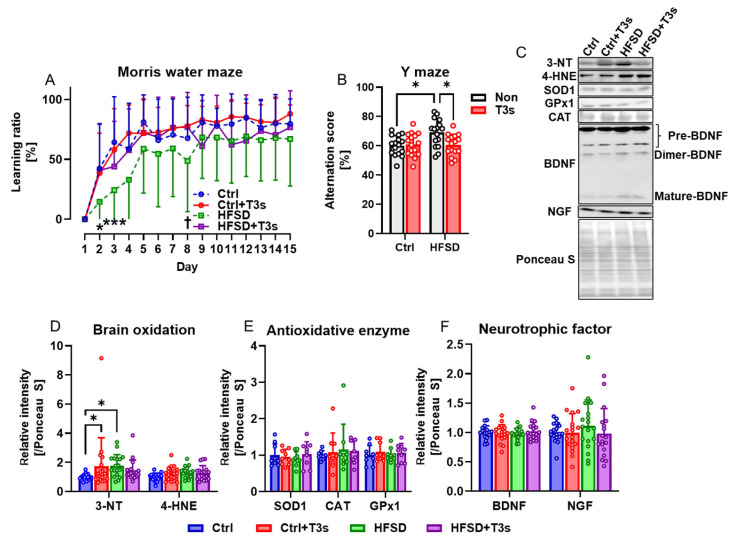
Obesity induced by feeding of HFSD decreases learning ability but improves short-term memory, while T3s treatment suppresses these changes. (**A**) Learning ratio measured using the Morris water maze test (HFSD vs. Ctrl; * *p* < 0.05, *** *p* < 0.001. HFSD vs. HFSD + T3s; † *p* < 0.05. all experimental groups; *n* = 16). (**B**) Alternation score of Y-maze as an index of short-term memory (* *p* < 0.05, all experimental groups; *n* = 16). (**C**) Representative images of Western blot. (**D**–**F**) Relative protein expression levels were measured in the Cortex (* *p* < 0.05. 3-NT, 4-hydroxynonenal (4-HNE), BDNF, NGF; *n* = 18 in all groups, SOD1, CAT, GPx1; *n* = 9 in all groups). All data are shown as the mean ± SD.

The Morris water maze and Y-maze test were performed to examine the influence of obesity on cognitive function in mice. The learning ability of HFSD-fed mice was significantly decreased compared to Ctrl-fed mice, but treatment with T3s significantly improved learning ability (Figure 2A). By contrast, the short-term memory of HFSD-fed mice was significantly greater than that of Ctrl mice, as determined by the Y-maze test, but co-treatment with T3s suppressed these cognitive changes (Figure 2B). 

Based on the free radical theory of aging, the level of brain oxidation was measured by Western blotting (Figure 2C,D). The results showed that protein oxidation was accelerated by HFSD feeding, and no significant difference was found between Ctrl + T3s and HFSD + T3s. However, there were no significant differences in antioxidant enzyme expression (Figure 2E). The expression of neurotrophic factors, which play a significant role in cognition, was also assessed using Western blotting. The relative expression level of NGF tended to (not significant) be higher in the brain of HFSD-fed mice than Ctrl mice (Figure 2F).

### 2.3. Respiratory Metabolism and Surface Temperature around the Scapula

**Figure 3:**
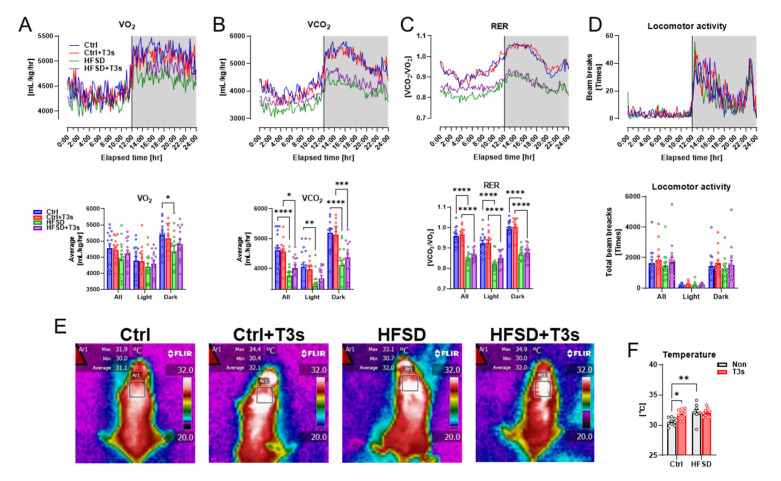
Feeding of HFSD alters respiratory metabolism and temperature near the scapula. (**A**–**D**) Indices of respiratory metabolism ((**A**), oxygen consumption; (**B**), carbon dioxide production; (**C**), volume ratio of VO_2_ and VCO_2_; (**D**), locomotion). The upper graphs show the changes for 24 h (white background during light; gray background during dark). The lower graphs show the average or total value for 24 h and light or dark period (* *p* < 0.05, ** *p* < 0.01, *** *p* < 0.001, **** *p* < 0.0001, all experimental groups and all parameters; *n* = 15). (**E**) Representative infrared images. (**F**) Quantification of the temperature around the scapula (* *p* < 0.05, ** *p* < 0.01, all experimental groups; *n* = 9). All data are shown as the mean ± SD.

As brain oxidation was enhanced in HFSD mice, the oxygen consumption volume was measured using a respiratory quotient device. Respiratory indices of HFSD and HFSD + T3s mice were significantly decreased compared to control mice without a reduction in locomotion (Figure 3A–D).

Treatment with T3s did not alter obesity indices such as body weight or serum parameters in HFSD-fed mice. The temperature around the scapula, which plays an important role in energy metabolism, was evaluated in each mouse group. The temperature around the scapula of T3s-treated mice was significantly higher than that of Ctrl mice. Additionally, feeding of an HFSD also significantly increased the temperature compared to Ctrl mice (Figure 3E,F).

### 2.4. Analysis of the Cortex Proteome of HFSD-Fed and/or T3s-Treated Mice

To determine the protein change by HFSD and/or T3s, a label-free quantitative proteomic analysis of the mouse Cortex was performed. In total, 4630 proteins were identified (Table S3), and 4061 proteins with high FDR confidence were detected in all groups. Among them, those exhibiting significant changes in expression between the HFSD and Ctrl groups were analyzed further. We focused on differentially-expressed proteins between HFSD and Ctrl groups, which were not changed between HFSD + T3s and Ctrl groups (Table 1). Obesity induced by HFSD feeding increased eight proteins (E3 ubiquitin-protein ligase MIB1, Reticulophagy regulator 3, Coiled-coil domain-containing protein 50, F-box only protein 3, Secretagogin, 45 kDa calcium-binding protein, high mobility group nucleosome-binding domain-containing protein 5, and Olfactory marker protein) and decreased four proteins (ADP-ribosylation factor 2, Neuropilin-2, Ubiquitin-conjugating enzyme E2 D1, and Histone–lysine N-methyltransferase STED1A) compared to Ctrl. These changes were significantly prevented in T3s-treated mice.

## 3. Discussion

Previously, we reported anti-obesity effects of T3s, such as attenuation of body weight gain, fatty liver development, and serum cholesterol increases in mice fed a high-fat diet (HFD) [22,26]. However, the anti-obesity effects of T3s in other obesity model mice remain unclear. To evaluate the anti-obesity effects of T3s in more detail, we fed mice an HFSD and measured various relevant parameters. Although T3s attenuated HFD-induced obesity in our previous studies, T3s did not suppress the HFSD-induced obesity observed in this study. A cell line model study reported that T3s reduce the enzymatic activity and protein expression of hydroxymethylglutaryl (HMG)-CoA reductase involved in cholesterol synthesis [27], but in the present study, there were no changes in serum cholesterol concentration in the presence or absence of T3s. This result indicates that the attenuating effect of T3s on serum cholesterol levels in HFD-fed mice is not mediated by HMG-CoA reductase. Although an arresting effect of T3s on the adipocyte cell cycle was observed in a previous study [28], T3s did not alter the weight of adipose tissues in HFSD-induced obesity model mice. Thus, results for HFD and HFSD model mice were contradictory. Several anti-obesity strategies have been reported that involve attenuating fat absorption from the small intestine [29,30]. To our knowledge, although there are no reports on the effects of T3s on fat absorption, T3s may inhibit the absorption of fat from the small intestine, but not sucrose. However, in the present study, we did not perform a test to evaluate the fat absorption capacity, such as an oral fat tolerance test. Further study is thus needed to elucidate the mechanism of the anti-obesity effect of T3s.

To clarify the relationship between HFSD-induced obesity and cognitive dysfunction, learning ability and short-term memory was assessed using the Morris water maze test and Y-maze test. The learning ratio of HFSD-fed mice was significantly lower than that of Ctrl mice only in the 2 and 3 days, but co-treatment with T3s inhibited this decline. These results suggest that HFSD does not decrease the absolute value of the learning function but delays learning speed. While there is much evidence on oxidation of adipose tissue and liver in obesity, oxidative balance in obese brain is poorly understood. We assessed the expression of 3-NT and 4-HNE in the brain using Western blotting. These analyses revealed that HFSD feeding significantly increased brain oxidation levels compared to Ctrl mice. Several studies have demonstrated that exposure of culture medium to oxidative stress induces neurodegeneration in some neuronal cell lines, such as Neuro2a, PC12, and SH-SY5Y [31,32,33]. Other studies have reported that increased oxidative stress in the brain leads to cognitive decline in rodent models, such as rats and mice [34,35]. Our results suggest that HFSD-induced obesity induces learning dysfunction via the enhancement of oxidative stress in the brain. We previously found that dietary T3s reach the brain [22]. Once in the brain, T3s may exert antioxidant effects that protect neurons directly rather than indirectly (Figure S1). T3s are metabolized in the living body, and these metabolites have physiological functions, but we have not measured the amount of metabolites. The effects of the metabolites on brain oxidation are unclear in this study.

In contrast to these results, short-term memory assessed using the Y-maze test was significantly better in the present study in HFSD-fed mice than in Ctrl mice. Moreover, NGF expression was nominally higher (but not significantly) in the brains of HFSD-fed mice compared with Ctrl mice. It is possible that this discrepancy may be related to the different cognitive parameters measured by the Morris water maze and Y-maze tests. The Morris water maze test assesses longer-term learning ability compared to the short-term memory assessment by the Y-maze test. The Y-maze test, by comparison, is primarily used to assess short-term memory. Oxidative stress may potentially impair learning ability but not short-term memory, and NGF may be involved in short-term memory. However, to elucidate the detailed mechanisms of how this discrepancy happens, further study is needed.

To elucidate the cause of brain oxidation in HFSD-fed mice, oxygen consumption was measured using whole-body analysis. Oxygen consumption was significantly lower in HFSD-fed mice than in Ctrl mice, and there was no difference in oxygen consumption in the presence or absence of T3s. As oxygen consumption did not differ, but the expression of 3-NT was increased in the brain of HFSD-fed mice, the reactive oxygen species (ROS) production ratio may be increased in HFSD-fed mice relative to Ctrl mice consuming the same volume of oxygen. It will be necessary to measure ROS in the brain of HFSD-fed mice using a technique such as electron spin resonance. 

The temperature around the scapula was measured as an index of thermogenesis in brown adipose tissue. Mice fed the Ctrl + T3s and HFSD exhibited significantly increased temperature in that area compared with Ctrl mice. However, there was no significant difference in the temperature of mice in the HFSD and HFSD + T3s groups. This result was consistent with the lack of a difference in body weight between HFSD and HFSD + T3s mice. 

To check the protein change associated with the consumption of the HFSD, a proteomic analysis was performed to identify proteins that were upregulated or downregulated in HFSD-fed mice. As a high proportion of proteins in Table 1 are involved in ubiquitination, we focused on these proteins.

E3 ubiquitin-protein ligase (MIB1) has been reported that it is associated with Notch signaling [36]. While it was reported that MIB2, which is a paralog of MIB1, directly interacts with B-cell CLL/lymphoma 10 (BCL10) and activates inflammatory regulator NF-kappa B, MIB1 does not interact with BCL10 [37]. As the changes of Notch signaling have been reported in AD brain, the increase of MIB1 in HFSD may be involved in obesity-induced cognitive changes [38,39]. Ubiquitin-conjugating enzyme E2 D1 (UBE2D1) was downregulated by HFSD feeding, but this downregulation was reversed by treatment with T3s. It has been reported that UBE2D1 attaches ubiquitin to other proteins, such as p53, which is a short-lived protein. UBE2D1 is involved in the ubiquitination of p53, which in turn induces p53 degradation [40]. Activation of p53 leads to apoptosis induction [41]. Consumption of an HFSD may induce apoptosis in neurons via UBE2D1 downregulation. Although this study found that UBE2D1 is downregulated in the brain of HFSD-fed mice, any associated changes in apoptosis or p53 activation remain unclear. F-box only protein 3 is one subunit of E3 ligase and has roles in many cellular processes via protein ubiquitination [42]. In this study, some ubiquitination-related proteins were altered by HFSD feeding. Additionally, although there are many papers on the relationships between cognitive function and ubiquitination, the detailed relationships between obesity and cognitive dysfunction and these proteins are unclear [43,44,45]. Excepting ubiquitination-related proteins, other proteins, for instance, secretagogin (relates to stress response) and histone–lysine N-methyltransferase (related to histone modification), were identified as the proteins changed by HFSD feeding. Further study is thus needed to elucidate the mechanism by which the HFSD impairs cognitive function.

## 4. Materials and Methods

### 4.1. Animals

All animal experiments were performed after approval by the Animal Protection and Ethics Committee of Shibaura Institute of Technology (Approval number #21002, Approval date, 26 August 2021). Three-week-old C57BL/6 male mice were purchased from Sankyo Labo Service Corp. Inc. (Tokyo, Japan) and acclimated to our animal housing conditions (room temperature; 22 ± 2 °C, 12 h light/dark cycle) for 1 week. A HFSD (#D12327, 40% kcal from fat and sucrose) and Ctrl against the HFSD (#D12324, 70% kcal from corn starch and maltodextrin, 10% kcal from fat) were purchased from Research Diets Inc. (New Brunswick, NJ, USA) (Figure 1A). The detailed nutrient composition of each diet is shown in Table S1. To assess the effects of T3s, 100 g of both the Ctrl and HFSD was fortified with 50 mg of T3-mix (Mitsubishi Chemical Corp., Tokyo, Japan), and the diets were named as Ctrl + T3s and HFSD + T3s, respectively. The body weight of each mouse was measured once per week. All mice were provided food and water ad libitum, and the weight of food and water intake over 24 h was also measured once per week.

### 4.2. Tissue and Serum Collection

After 16 h fasting, the liver, Cortex, epididymal, and perirenal fat were collected and weighed as soon as possible after removal. Blood was sampled from the inferior vena cava and centrifuged at 4000 rpm for 20 min. The supernatants were collected as serum samples and used for biochemical analysis.

### 4.3. Serum Cholesterol, Triglyceride and Glucose Concentrations

T-CHO, TG, and glucose concentrations in the serum were measured by an animal inspection service (Oriental Yeast Co., Ltd., Tokyo, Japan).

### 4.4. Chemical Regents

All reagents were purchased from FUJIFILM Wako Pure Chemical Corp. (Osaka, Japan) or Sigma-Aldrich Corp. (St. Louis, MO, USA) unless otherwise indicated.

### 4.5. Behavioral Assessment

#### 4.5.1. Morris Water Maze Test

Learning ability was evaluated using the Morris water maze test, as described previously [22]. Before starting trials, mice were handled for 3 min by the experimenter and allowed to swim for 1 min in the apparatus (Muromachi Kikai Co., Ltd., Tokyo, Japan) without the goal for 3 consecutive days. During trials, mice were allowed to swim freely for a maximum of 1 min per day in the apparatus with the goal, and we considered an arrival as a mouse being on the goal for longer than 1 s. The trials were performed for 15 consecutive days and recorded using a web camera. The recorded trials were analyzed using ANY-maze software (ver. 4.98; Stoelting Co., Wood Dale, IL, USA) to measure the time required by each mouse to reach the goal. The learning ratio of day X was then calculated as the goal time on day X divided by the goal time on day 1, which was subtracted from 1 and multiplied by 100. If the calculated ratio was less than zero, the ratio was considered zero. 

#### 4.5.2. Y-Maze Test

A Y-maze apparatus (Muromachi Kikai Co., Ltd.) was used to assess short-term memory, as described previously [26]. Briefly, spontaneous alternation testing was performed by placing a mouse in the apparatus and recording its movement for 10 min. The recorded movement of the mouse was analyzed, and the number of total arm entries and alternation scores were calculated using ANY-maze software (Stoelting Co.).

#### 4.5.3. Western Blotting

Extraction of protein from the Cortex and Western blotting were performed, as described previously [22]. A total of 20 μg of extracted protein from each sample was separated on 12% polyacrylamide gels and then transferred onto nitrocellulose membranes. To block non-specific binding, the membranes were incubated with 2% skim milk in Tris-HCl buffered saline containing 0.1% Tween 20 (TBS-T). The membranes were then incubated with primary antibodies overnight at 4 °C. The primary antibodies are described in Table S2. After reaction with the primary antibody, the membranes were treated for 1 h with anti-mouse or rabbit IgG HRP antibody (Promega Corp., Madison, WI, USA) at 1:4000 dilution. Finally, the membranes were exposed to Immobilin Western Chemiluminescent HRP substrate (Merck, KGaA., Darmstadt, Germany), and chemiluminescent signals were detected using a LAS-3000 (FUJIFILM Corp.) and analyzed using Image Quant software (ver. 8.2.0.0; Cytiva, Tokyo, Japan). Western blotting experiments were performed 1–3 times per sample.

### 4.6. Energy Metabolism and Locomotor Activity

Oxygen consumption and carbon dioxide production were measured every 10 min using an Oxymax System (Columbus, OH, USA). Locomotor activity during energy metabolism measurements was assessed as the number of beam crossings using the ACTIMO system (SHINFACTORY, Fukuoka, Japan).

### 4.7. Thermography

Infrared images were acquired using an infrared camera (Teledyne FLIR, LLC., Wilsonville, OR, USA). The data were then analyzed using FLIR Tools software (ver.2.0; Teledyne FLIR, LLC.) to determine the temperature around the mouse scapula. 

### 4.8. Sample Preparation for Proteomic Analysis

For protein extraction, Cortexes were homogenized in RIPA buffer (*w*/*v*: 1:9) and centrifuged at 13,000 rpm for 20 min. The supernatants were collected, and the protein concentration was measured using Pierce 660 nm Protein Assay Regent (Thermo Fisher Scientific Inc., Waltham, MA, USA). A Multiskan GO (Thermo Fisher Scientific Inc.) was used to read the absorbance. One hundred micrograms of protein was precipitated using a ProteoExtract Protein Precipitation kit (Merck KGaA) and dissolved in 50 mM NH_4_CO_3_ containing 0.1% RapiGest SF (Waters Corporation, Milford, MA, USA). The protein concentration was measured using Pierce 660nm Protein Assay Regent with Ionic Detergent Compatibility Reagent (Thermo Fisher Scientific Inc.). For protein digestion, 10 μg of protein was reduced using 5 mM dithiothreitol at 60 °C for 30 min, alkylated using iodoacetamide at room temperature for 30 min in the dark, and digested with 0.2 μg of trypsin (1:50 enzyme-to-protein ratio) at 37 °C overnight. The digested sample was acidified using 10% trifluoroacetic acid (final 0.5%) and incubated at 37 °C for 30 min to stop the digestion and decompose RapiGest SF. Then, the sample solutions were desalted using GL-Tip SDB (GL Sciences Inc., Tokyo, Japan). The eluate was evaporated in vacuo to dryness using a centrifugal evaporator (TOKYO RIKAKIKAI Co., Ltd., Tokyo, Japan) and reconstituted in 2% acetonitrile (MeCN) containing 0.1% formic acid (FA) at a concentration of 0.5 μg/μL.

### 4.9. Liquid Chromatography–Tandem Mass Spectrometry (LC-MS/MS) Analysis

An Ultimate 3000 RSLCnano system (Thermo Fisher Scientific Inc.) coupled to a Q Exactive hybrid quadrupole-Orbitrap mass spectrometer (Thermo Fisher Scientific Inc.) was used for LC-MS/MS analysis of tryptic peptides. PepMap Neo (0.3 mm × 5 mm, 5 μm, Thermo Fisher Scientific Inc.) and NANO HPLC Capillary Column (0.075 mm × 12 cm, 3 μm, Nikkyo Technos, Tokyo, Japan) were used as the trap and analytical column, respectively. Peptide separation was performed in water containing 0.1% FA (mobile phage A) and MeCN containing 0.1% FA at a flow rate of 300 nL/min. The concentration of mobile phase B was changed as follows during peptide separation: 0–3 min, 2%; 3–123 min, 2–40%; 123–125 min, 40–95%; 125–135 min, 95%; 135–137 min, 95–2%; 137–150 min, 2%. The sample injection volume was 2 μL corresponding to 1 μg protein. 

The mass spectrometer was operated in data-dependent acquisition (DDA) mode. All mass spectra were acquired with the following settings: spray voltage, 2.0 kV; capillary temperature, 275 °C; S-lens RF level, 50; polarity, positive ion; resolution, 70,000; automatic gain control (AGC) target, 3 × 10^6^; maximum ion injection time (IT), 100 ms; scan range, *m*/*z* 350–1500. The parameters of DDA mode were as follows: resolution, *m*/*z*; normalized collision energy, 27; dynamic exclusion, 15 s; charge exclusion, unassigned, 1, 8, >8.

LC-MS/MS data were analyzed to perform the identification of proteins and label-free quantification (LFQ) of proteins using Proteome Discoverer 2.4 software (Thermo Fisher Scientific Inc.). Parameters for protein identification were as follows: search engine, Sequest HT; parent mass tolerance, 10.0 ppm; fragment mass tolerance, 0.02 Da; enzyme, trypsin (full); max missed cleavage sites, 2; static modification, carbamidomethyl (Cys, +57.021 Da); dynamic modification, oxidation (Met, +15.995 Da); protein database, Mus musculus (SwissProt, reviewed, 17,025 sequences); FDR confidence, high < 0.01, medium < 0.05. Parameters for LFQ were as follows: precursor abundance based on the area; normalization mode, total peptide amount; protein ratio calculation, pairwise ratio based; hypothesis test, *t*-test (background-based).

The selection method of proteins in Table 1 is below. Firstly, the proteins whose FDR confidence levels were high and which were detected in all groups were listed. Secondly, the listed proteins were narrowed down by the abundance ratio of HFSD, which was <0.5 or >2, and the *p*-value between Ctrl and HFSD + T3s > 0.05.

### 4.10. Statistical Analysis

Data are expressed as the mean ± SD. A two-way analysis of variance followed by the Tukey–Kramer’s test was used for multiple comparisons; *p* < 0.05 was considered statistically significant. All statistical analyses were performed using GraphPad Prism 9 (Graph Pad Software, San Diego, CA, USA).

## 5. Conclusions

In conclusion, this study revealed that HFSD-induced obesity leads to a decline in learning ability as measured by the Morris water maze test, and treatment with T3s suppresses HFSD-induced cognitive changes. One reason for the HFSD-induced cognitive decline was revealed to be enhanced oxidation in the brain. However, how the HFSD induces brain oxidation remains unclear. Further study is needed to clarify the mechanism by which the HFSD accelerates oxidation in the brain. 

While it has been reported that T3s attenuate HFD-induced obesity, this study revealed that the anti-obesity effects of T3s could not be exerted in HFSD-induced obesity model mice [19,22,26]. When evaluating the anti-obesity effects of T3s, it is thus important to carefully determine the cause of obesity.

## Figures and Tables

**Table 1:** Identification of Cortex proteins for which expression is affected by experimental diets. * *p* < 0.05.

		Abundance Ratio		
Accession	Description	HFSD/Ctr1	HFSD + T3s/Ctrl	HFSD + T3s/HFSD
P61080	Ubiquitin-conjugating enzyme E2 D1	0.39 *	1.2	3.1 *
Q8BSL7	ADP-ribosylation factor 2	0.44 *	0.70	1.5
E9PYH6	Histone-lysine N-methyltransferase SETD1A	0.46 *	0.85	1.9 *
O35375	Neuropilin-2	0.46 *	0.92	1.8 *
Q9CQV4	Reticulophagy regulator 3	2.0 *	1.4	0.69 *
Q91WD9	Secretagogin	2.0 *	1.1	0.55 *
Q810U5	Coiled-coil domain-containing protein 50	2.1 *	1.7	0.87
Q9DC63	F-box only protein 3	2.1 *	1.0	0.46 *
Q80SY4	E3 ubiquitin-protein ligase MIB1	2.2 *	1.2	0.55 *
Q64288	Olfactory marker protein	2.5 *	0.86	0.34 *
Q61112	45 kDa calcium-binding protein	2.6 *	1.6	0.66 *
Q9JL35	High mobility group nucleosome-binding domain-containing protein 5	3.0 *	1.5	0.49 *

## Data Availability

All data generated or analyzed during this study are included in this published article.

## Supplementary Materials

The following supporting information can be downloaded at: https://www.mdpi.com/article/10.3390/ijms25063561/s1.
