# Supplementary figures and images for: Tocotrienols Prevent the Decline of Learning Ability in High-Fat, High-Sucrose Diet-Fed C57BL/6 Mice
Source: Int J Mol Sci. 2024 Mar 21;25(6):3561. doi: 10.3390/ijms25063561 (PMC10971311; doi:10.3390/ijms25063561)

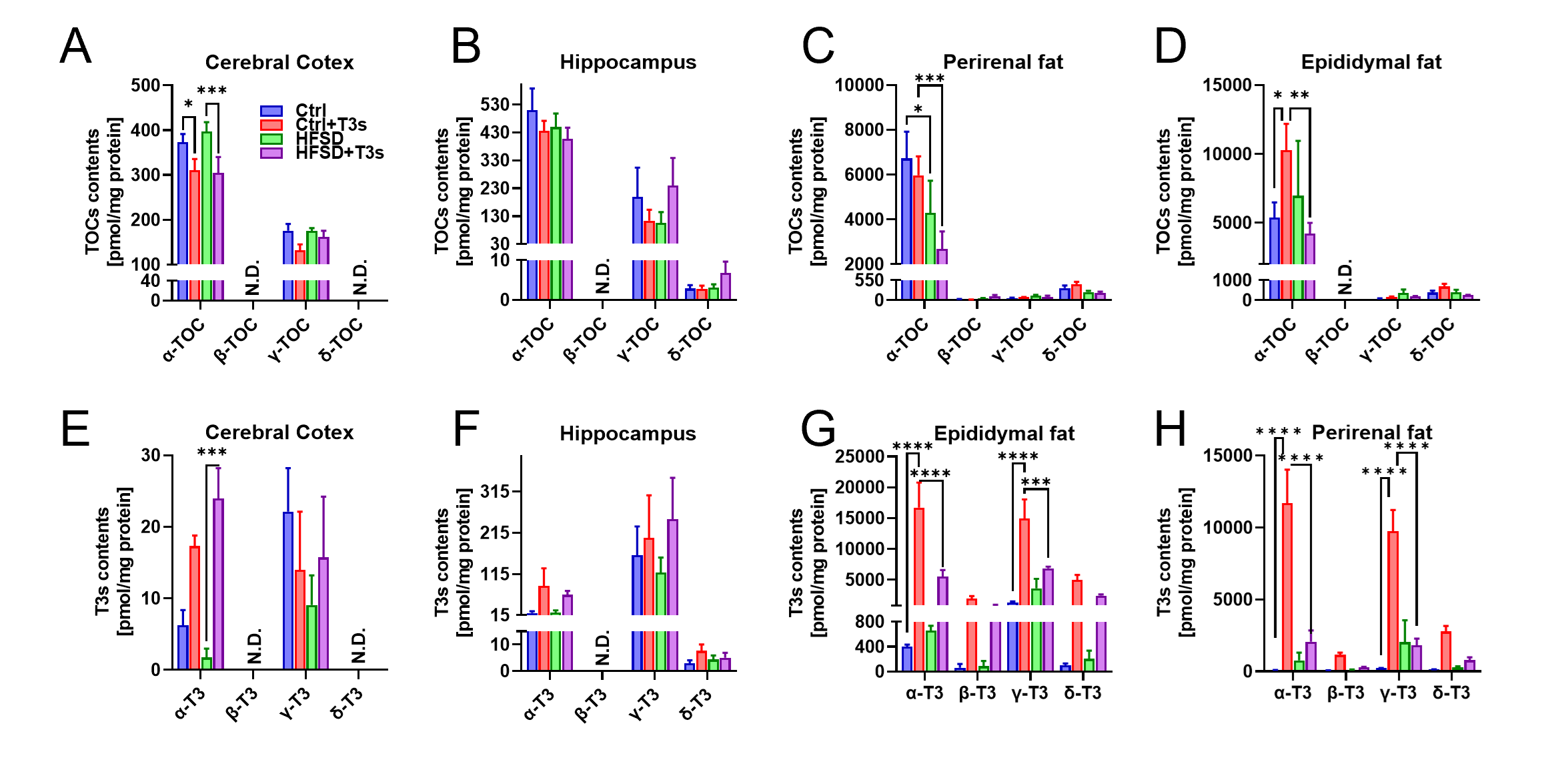

Supplement: Supplementary file 1 [file ijms-25-03561-s001.zip › Figure S1.tif]

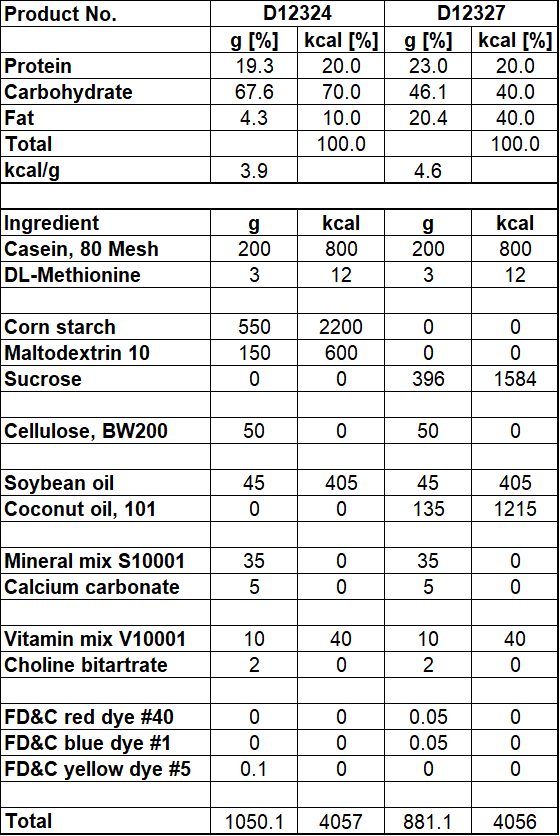

Supplement: Supplementary file 1 [file ijms-25-03561-s001.zip › Table S1.tif]

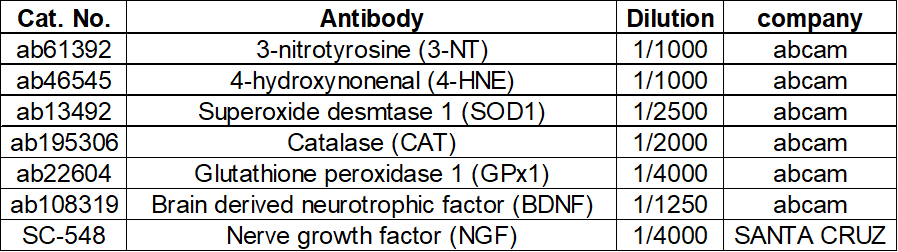

Supplement: Supplementary file 1 [file ijms-25-03561-s001.zip › Table S2.tif]
